# Herd Dominance Influences Dairy Cows’ Use of Heat Abatement Resources in a Silvopastoral System

**DOI:** 10.3390/ani15121791

**Published:** 2025-06-18

**Authors:** Matheus Deniz, Amanda Ribeiro Sena, Karolini Tenffen De-Sousa, Frederico Márcio Corrêa Vieira, Estevam Rodrigues de Souza, Maria José Hötzel, João Ricardo Dittrich

**Affiliations:** 1 Grupo de Estudos em Bovinos Leiteiros, Faculdade de Medicina Veterinária e Zootecnia, Universidade Estadual Paulista, Botucatu 18618-681, São Paulo, Brazil; ar.sena@unesp.br (A.R.S.); karoltenffen10@hotmail.com (K.T.D.-S.); estevam.rodrigues@unesp.br (E.R.d.S.); 2 Grupo de Estudos em Biometeorologia, Universidade Tecnológica Federal do Paraná, Dois Vizinhos 85660-000, Paraná, Brazil; fredericovieira@utfpr.edu.br; 3 Programa de Pós-Graduação em Zootecnia, Faculdade de Medicina Veterinária e Zootecnia, Universidade Estadual Paulista, Botucatu 18618-681, São Paulo, Brazil; 4 Laboratório de Etologia Aplicada e Bem-Estar Animal, Universidade Federal de Santa Catarina, Florianópolis 88034-000, Santa Catarina, Brazil; maria.j.hotzel@ufsc.br; 5 Programa de Pós-Graduação em Zootecnia, Universidade Federal do Paraná, Curitiba 80035-050, Paraná, Brazil; dittrich@ufpr.br

**Keywords:** animal welfare, biometeorology, social behavior, thermal comfort

## Abstract

The social category of dairy cows within a herd may influence their access to essential heat abatement resources. We evaluated how dominant, intermediate, and subordinate cows used shade and water in a silvopastoral system. Thirty-nine lactating Jersey cows were observed in a replicated study. Cows had unrestricted access to natural shade and a water trough. Microclimatic conditions and cow behaviors were recorded, and social rank was determined. Sunny areas promoted potential thermal discomfort for the cows, with higher heat load (on average 580.7 W/m^2^) than shaded areas (on average 438.6 W/m^2^). Intermediate (IRR: 2.07) and subordinate (IRR: 1.63) cows were more likely (*p* < 0.001) to visit the water trough than dominant cows. The odds of drinking water decreased by 13% (*p* < 0.001) for each hour that passed throughout the day. In contrast, the odds of using shaded areas increased by 7% (*p* < 0.001) for each increase in hour unit. Although all cows used the shaded areas at some point, they generally spent more time standing than lying down in the shade. Social category and hour influenced (*p* < 0.001) the behaviors in the shaded areas, with dominant cows more likely to engage in lying behaviors such as idling and rumination. In conclusion, our study demonstrated that cows’ social category and thermal environment influenced the use of shade and drinking water. Although the overall pattern of resource use increased with rising thermal load, the proportion of use varied according to social category. Ensuring multiple or alternative resources may help meet the needs of cows across social ranks and improve thermal comfort during heat stress periods.

## Simple Summary

In this study, we investigated how the social category of cows within a herd can influence their use of shade and water, two important resources for coping with heat. We observed 39 Jersey cows in a pasture-based system with access to natural shade and water. Dominant cows used the shaded areas more often, while subordinate and intermediate cows visited the water trough more frequently. These findings suggest that social relationships among animals directly affect their thermal comfort, especially during hot periods. This study highlights the importance of providing ad libitum access to heat abatement resources to improve the thermal comfort of the herd. These results can encourage farmers to adopt more effective management practices that promote animal welfare in different production systems.

## 1. Introduction

The current climate change scenario calls for a remodeling of livestock systems. As global air temperatures rise, the number of hours and days cattle remain within their thermal comfort zone decreases [1,2]. Based on this, silvopastoral systems align with the growing need to provide heat abatement resources for animals while simultaneously addressing global demands to reduce greenhouse gas emissions and meet societal expectations for sustainable practices that ensure animal welfare [3]. As a form of agroforestry, these systems promote the diversification of agroecosystem components, such as soil, pasture, livestock, and trees, through varied designs, spacings, and combinations of tree and forage species [4,5], making them a versatile and adaptive strategy in the face of climate change. However, their successful implementation depends on regional constraints, including climate conditions and local socioeconomic and cultural factors [6]. Silvopastoral systems have shown positive outcomes in several countries (e.g., Canada [7], Mexico [8], Morocco [9], and Germany [10]), but the use of specific heat abatement resources, such as shade and water, by cattle within these systems remains underexplored.

Hot thermal conditions increase cows’ motivation to seek resources that help them lose heat to the environment [11], e.g., drinking water [12] and accessing shade [13] or ventilated areas [14]. However, the access of animals does not occur homogeneously, as dominant animals have priority access to available resources (food [15], water [16], and brush [17]). Differences in resource use may prevent some animals from meeting their nutritional needs, impairing their performance and ability to deal with environmental challenges. Thus, subordinate animals adopt behavioral strategies to satisfy their needs. For example, in free-stall housing, subordinate cows adjust their drinking behavior on hot days to avoid the drinkers during periods of high competition [12], and in compost-barns, subordinate cows are more likely to drink water in the hottest hours while dominant cows lie down in ventilated areas [18]. In pasture areas, subordinate heifers spend more time grazing, while dominant heifers spend more time eating grain [15]. Although some studies have addressed resource use in different housing systems, there is a lack of information about how cows of different social ranks use shade and water throughout the day in silvopastoral systems, which this study aimed to address. We predicted that dominant cows would have priority access to heat abatement resources, particularly during periods of greater thermal challenge. The aim of this study was to evaluate the use of two heat abatement resources, shade and water, throughout the hours of the day by different social categories in a group of dairy cows maintained in a silvopastoral system.

## 2. Materials and Methods

### 2.1. Animals, Management and Study Design

This experiment was carried out in the state of Paraná (25°26′41″ S, 49°11′33″ W) in Southern Brazil covering the four seasons between March 2020 and February 2021. The climate of the region is characterized as humid maritime temperate, Cfb [19]. At the farm, cows were kept permanently on pasture (763 kg/DM/ha with 16.1% crude protein in cold seasons and 1276 kg/DM/ha with 19.5% crude protein in hot seasons) and managed under the Voisin’s Rotational Grazing system [19]. The pasture composition in all paddocks comprised *Axonopus* spp., *Paspalum* spp., *Pennisetum* spp., *Cynodon* spp., *Trifolium* spp., and *Lolium* spp. with an average height of 25 cm and a 45-day resting period.

A total of 39 lactating Jersey cows (mean ± SD: 5.2 ± 2.5 years of age, 396.6 ± 42.0 kg body weight, 16.8 ± 3.6 L/day milk yield, and 144.4 ± 94.4 days in milk) were enrolled across the four seasons in a replicated study. Our study was conducted on a commercial farm, keeping the daily routine and existing infrastructure. In accordance with farm management practices, the group size was maintained at 22 cows per season; however, group composition was dynamic, as cows entered the group within 24 h after calving and left approximately 60 days before their expected calving date. In each season, data collection consisted of two experimental periods: Period 1, comprising two consecutive days during the optimal pasture regrowth phase [19]; and Period 2, comprising two consecutive days following the pasture rest phase. Rainy and cloudy days were avoided in both periods to ensure consistent environmental conditions.

During the experimental periods, all cows (22 cows per season) remained together in a silvopastoral system (paddocks with 2400 m^2^), with ad libitum access to natural shaded areas (~240 m^2^) and a single water trough (120 cm diameter, 60 cm high, and 500 L capacity). The water trough always remained in the sunny area. The natural shading was provided by trees (100 trees/ha) in a single row along the border fence. The silvopastoral system was implemented 10 years prior to the study and had approximately 20 trees per paddock in a northeast–southwest orientation (*Pyrus communis*, *Ligustrum japonicum*, *Pyrus calleriana*, *Zanthoxylum rhoifolium*, *Allophylus edulis*, and *Podocarpus lambertii*), with 30 m between rows and 4 m between trees. The trees’ height during the experimental period was on average 8 m; the average shaded area was 10.7 m^2^ per animal, which is sufficient for all animals [20].

We established the social hierarchy based on displacements at the feeder, following the method described by Deniz et al. [21]. Using the “socialh” package [22] in R [23], we constructed a sociometric matrix by season that recorded the number of wins and losses for each animal in displacement events against every other member of the group. The dominance value (DV) for each animal was then calculated in two steps, following the method developed by Kondo and Hurnik [24]. In the first step, the social position (Sij) of each individual i relative to individual j was calculated based on the number of wins and losses between them using the sociometric matrix. The value of Sij could be +1 or −1, depending on whether i wins or loses against j, respectively. When i = j (self-interaction), the value is zero. In the second step, the dominance value (Si) for each individual was calculated as the sum of its Sij values. Based on the DV, animals were classified into social hierarchy categories: subordinate (1), intermediate (2), or dominant (3). These categories were defined using the method proposed by Coimbra et al. [16], which divides the range between the highest (+X) and lowest (−Y) DV values, plus 1, by three (corresponding to the number of hierarchy levels).

### 2.2. Study Measurements

The data collection period (four days per season, resulting in a total of 16 days) was based on the experimental period used in several studies [25,26,27]. The experimental period was selected based on the weather forecast (www.accuwheather.com, accessed on 30 April 2025) predicting daytime without clouds. Cloudless days were a requirement to measure the intensity of cows’ use of the different heat abatement resources (shaded areas and water). Thermal environment and behavior data were recorded between the morning and the afternoon milking (8 h to 15 h), following standard farm management.

### 2.3. Thermal Environment

We recorded air temperature (AT, °C), relative humidity (RH, %), soil surface temperature (SST, °C), and black globe temperature (BGT, °C) with five minute intervals by four autonomous dataloggers (ADEFs; for details and validation, see Deniz et al. [28]). Two ADEFs were located with full sun exposure (sunny area—distant from the trees), and the other two were located in the shaded area, 2 m distance from the trees. The wind speed (WS) was measured using a thermo-anemometer (Model HM-833; 0.1–35 m/s scale; ±7% + 0.70 m/s precision; 0.01 m/s resolution). The variables AT, RH, WS, and BGT were measured at a height of 1.3 m from the ground, which corresponds to the height of the mass center of an adult Jersey cow. The SST was measured at the level of the soil under the pasture. Based on microclimatic measurements, we calculated the radiant heat load (RHL, W/m^2^) and black globe humidity index (BGHI). Additionally, we determined the accumulated value of thermal load (Acc) for the BGHI, which was calculated as proposed by Buffington et al. [29], using Equation (1). The values obtained indicated ≤74: thermal comfort situation; 75–78: warning; 79–84: danger; and ≥85: emergency [30].

BGHI = BGT + 0.36 × (AT − (100 − RH)/5 + 41.5(1)

where BGHI is the black globe humidity index, BGT is the black globe temperature (°C), AT is the air temperature (°C), and RH is the relative humidity.

The RHL was used to express the total radiation received directly and indirectly by the animals, obtained by Equation (2) proposed by Esmay [31].

RHL = σ × (Tm^2^) (2)

where σ is the Stefan–Boltzman constant, 5.67 × 10^−8^ K4 (W/m^2^), and Tm is the mean radiant temperature (W/m^2^).

The accumulated value of thermal load (Acc) was calculated as proposed by Volpi et al. [32], using Equation (3). The Acc always results in a value of +Y, 0, or −Y. Thus, if the measured value is greater than the threshold, this results in a positive difference (+Y), i.e., the animal is receiving heat from the environment. If the measured value is equal to the threshold, this results in zero, i.e., the animal is in a thermal-neutral condition, and if the measured value is less than the threshold, this results in a negative difference (−Y), i.e., the animal is losing heat to the environment.

Acc = (threshold value − measured value) × (−1)(3)

### 2.4. Animal Behavior

For behavior evaluation, each cow was identified with a number painted on the lumbar area with commercial animal marking crayon. The behaviors of grazing, rumination, and idling, as defined by Bica et al. [15], along with postures (standing and lying down), were directly recorded through scan sampling at 10 min intervals following the methodology proposed by Altman [33]. Additionally, at each scan, we registered the area (shade or sun) in which the cows were performing the behavior. A cow was considered in the shaded area when more than 50% of her body was under the shade of a tree [19], and water intake was recorded when her lips were immersed in water, with neck movements indicating ingestion [16]. The cows were observed for experimental data collection from 8:00 to 15:00. After this period, all cows were moved for milking. During the interval between experimental periods, the cows occupied other paddocks on the farm to allow regrowth of pasture in the experimental paddocks. All behavioral observations were conducted by two researchers who had been previously trained. An inter-observer reliability test [34] was carried out over two days (3 h/day) in a pilot study to ensure consistency between observers. During the pilot study, all possible behaviors were exhibited, and the observers were trained until they achieved a reliability score of ≥80% [34], using the first author (M.D.) as the reference.

### 2.5. Statistical Analysis

All descriptive and confirmatory analyses were conducted in R using RStudio (version 4.3.2; R Core Team, [23]). The dataset was structured with each observation comprising local microclimate variables, bioclimatic indicators, and frequency of behaviors summarized by hour, date, and area (shaded or sunny). For microclimate variables and bioclimatic indicators, one observation per hour per day was generated, while for frequency of behaviors, one observation per animal per hour per day was generated. Additionally, for descriptive analysis and figures generation, the behaviors were weighted according to the number of cows in each social category (Table 1) to eliminate the numerical discrepancy.

Confirmatory analyses were conducted using Generalized Linear Mixed Models (GLMMs) with a 95% confidence interval. All models were adjusted through the maximum likelihood test approximation method with the statistical package lme4 [35], and the confidence intervals were estimated using Type II Wald chi-square tests. Model fit was evaluated by inspecting the residual graphs and comparing the Akaike information criterion (AIC), where the model with a lower AIC information criterion was deemed to have a better fit. The normality of the random factors was given by quartile plot means with a confidence interval of 95%. Details of model for each variable are presented below.

For each thermal environment variable, a Generalized Linear Mixed Model (GLMM) with a Gamma distribution was fitted. These models included area (shade and sun) and hours (levels 1 to 7) as fixed effects, with day nested within season as a random effect.

For each behavior evaluated, a GLMM with a Poisson distribution was fitted. These models included social category (dominant, intermediate, or subordinate), area (shade or sun), hours (levels 1 to 7), and BGHI category (thermal comfort, warning, danger, or emergency) as fixed effects, with animal and day nested within season as random effects. For interpretation purposes, the incidence rate ratio (IRR) was used. The IRR represents the odds of a given event occurring in relation to the reference category (social hierarchy = dominant; areas = shade; and BGHI = thermal comfort).

## 3. Results

### 3.1. Thermal Environment

**Figure 1:**
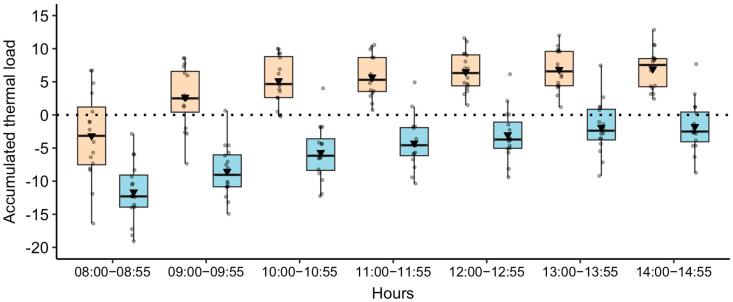
Boxplot of accumulated thermal load values over the hours of evaluation. Positive values indicate heat gain from the environment and negative values indicate heat loss to the environment, under shaded (blue box) and sunny (peach box) conditions. The black triangle represents the mean, while the solid black line represents the median. The box limits correspond to the 25th and 75th percentiles (first and third quartiles). Gray points represent individual observations, jittered using the ‘geom_jitter’ function [36].

Microclimatic variables and thermal comfort indicators differed (*p* < 0.05) according to the area (shaded and sunny) and hours (Table 2). Sunny areas promoted potential thermal discomfort for the cows with higher heat load (on average 580.7 W/m^2^) than shaded areas (on average 438.6 W/m^2^). Across all evaluated days of the seasons, the BGHI was above 74 for 85% of the hours in the sunny area. During the spring, there were two days with four consecutive hours where the BGHI exceeded 74 in the shaded area. Similarly, during the summer, this occurred for three consecutive hours on two separate days in the shaded area. Regardless of the season, the accumulated value of thermal load in sunny areas exceeded the critical threshold in the second hour of evaluation (Figure 1).

### 3.2. Animals Behavior

**Figure 2:**
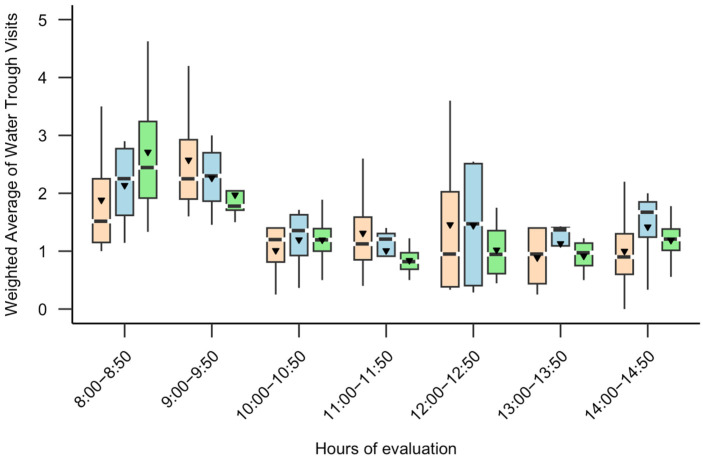
Boxplot of weighted average of number of water trough visits of different social categories (dominant: peach box, intermediate: blue box and subordinate: green box) throughout the hours of evaluation. The black triangle represents the mean, while the solid black line represents the median. The box limits correspond to the 25th and 75th percentiles (first and third quartiles).

The frequency of visits to the water trough was influenced by both social category (*p* < 0.001) and hours (*p* < 0.001). Intermediate cows (IRR: 2.07; 95% CI: 1.74–2.46; z = 8.29; *p* < 0.001) and subordinate cows (IRR: 1.63; 95% CI: 1.36–1.96; z = 5.33; *p* < 0.001) were more likely to visit the water trough compared to dominant cows. Additionally, the odds of drinking water decreased by 13% with each additional hour of observation (95% CI: 0.84–0.91; z = −8.04; *p* < 0.001). Descriptively, all cows visited the water trough during the experimental period, and the highest frequencies of visits (Figure 2) occurred during the first two hours of observation (8:00–8:50: 208 events; 9:00–9:50: 195 events). The average frequency of visits varied among social categories: dominant cows visited the trough 28.4 times per hour (range: 18–51), intermediate cows 56.7 times per hour (range: 38–83), and subordinate cows 45.4 times per hour (range: 28–89).

**Figure 3:**
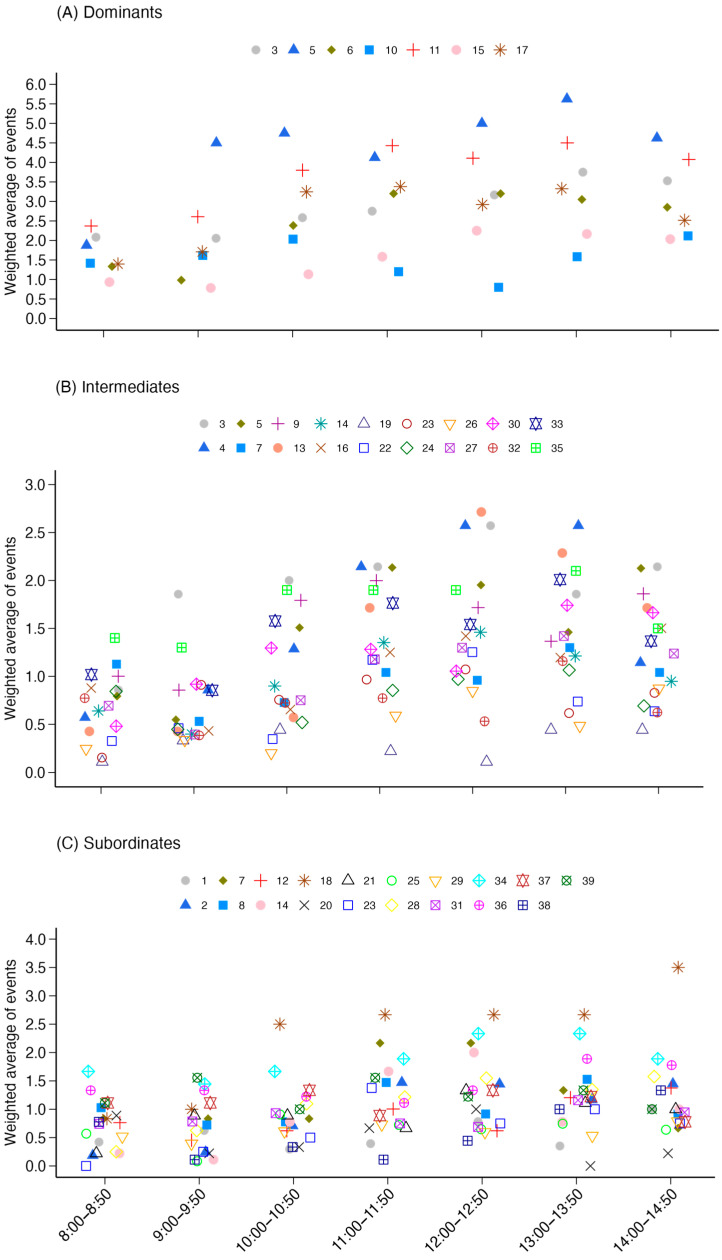
Individual weighted average of events of use of the shaded areas by social category (**A**) dominants, (**B**) intermediates, and (**C**) subordinates) throughout the hours of evaluation. Each cow is represented by a symbol and color. Symbols were jittered using the ‘geom_jitter’ function [36].

Descriptively, all cows used the shade during the experimental period (Figure 3), except one subordinate cow that did not access the shade at any time. No statistical inference was provided for these outcomes because we did not have any a priori hypotheses. When in shaded areas, cows spent on average more time standing (65.8%; range: 18.2–100%) than lying down (34.2%; range: 0–80.1%).

**Figure 4:**
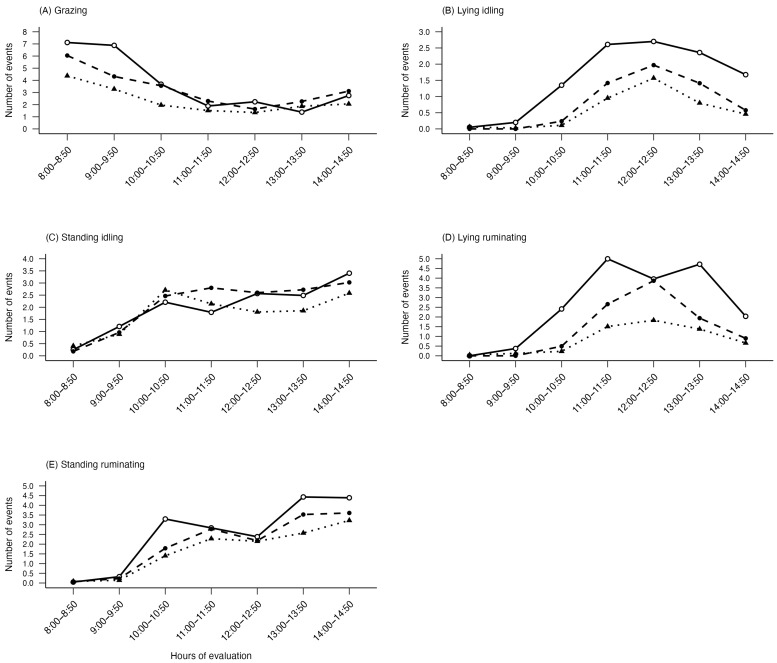
Weighted average of number of events by behaviors (**A**) grazing, (**B**) lying idling, (**C**) standing idling, (**D**) lying ruminating, and (**E**) standing ruminating in the shaded areas by social category (dominant, intermediate and subordinate) throughout the hours of evaluation. The solid lines with white circles represent dominant cows, dashed lines with black circles represent intermediate cows, while dotted lines with triangles represent subordinate cows.

Social categories (*p* < 0.001) and hours (*p* < 0.001) influenced behaviors performed within the shaded areas. Regardless of the behavior, the intermediate (IRR: 0.80; 95% CI: 0.76–0.84; z = −8.02; *p* < 0.001) and subordinate (IRR: 0.66; 95% CI: 0.63–0.70; z = −13.78; *p* < 0.001) cows were less likely to use the shaded areas than dominant cows. Descriptively, the time spent in the shade varied across social categories (Figure 4). On average, each dominant cow was observed in the shaded areas for 12.26 events (range: 4–22.5), each intermediate cow was observed in shaded areas for 9.92 events (range: 1–21), and each subordinate cow was observed in shaded areas for 7.35 events (range: 0–21).

When in shaded areas, intermediate and subordinate cows were less likely to engage in lying idling (I-IRR: 0.6; CI: 0.51–0.71; z = −6.24; *p* < 0.001; S-IRR: 0.68; CI: 0.58–0.81; z = −4.62; *p* < 0.001) and lying rumination (I-IRR: 0.64; CI: 0.56–0.72; z = −7.03; *p* < 0.001; S-IRR: 0.55; CI: 0.49–0.64; z = −8.61; *p* < 0.001) compared to dominant cows. However, social categories did not influence standing behavior (rumination: CI: 0.82–1.06; z = 0.80; *p* = 0.42 and idle: CI: 0.95–1.25; z = 1.26; *p* = 0.21).

Regardless of the social category, cows were 25% more likely (95% CI: 0.69–0.95; z = −2.593; *p* = 0.009) to use the shaded areas during the danger category of BGHI than during the thermal comfort category of BGHI. The lowest frequency of shaded used was observed in the first two hours of observation (8:00–8:50: 879 events and 09:00–09:50: 858 events). The odds of using shaded areas increased by 7% (95% CI: 1.06–1.09; z = 13.65; *p* < 0.001) for each increase in hour unit.

## 4. Discussion

Our study showed that cows’ access to shade and water is influenced by social category, hours and thermal environment. Although the overall patterns of shade and water use were similar among all animals throughout the hours of evaluation, the proportion of resource use varied by social category. Intermediate and subordinate cows visited the water trough more frequently, whereas dominant cows consistently occupied shaded areas during observation periods. Furthermore, when intermediate and subordinate cows were present in shaded areas, they were less likely to lie down, possibly indicating less effective use of the resource for resting and thermoregulation. On the other hand, dominant cows may have used the shaded areas more efficiently for heat dissipation via sensible heat loss, as these areas exhibited lower thermal comfort index values and cooler soil surface temperatures. Social hierarchy influences not only access to but also the quality of resource used, with dominant cows typically gaining priority and longer access durations [15]. These findings underscore the importance of providing multiple heat mitigation resources in sufficient quantity to meet the needs of all cows, regardless of their social category. Cows do not simply choose between shade or water; instead, they dynamically adjust their use of both, influenced by social interactions and thermal conditions.

Patterns of shade and water use offer insights into when these resources are most valued by the animals, once cows make choices and alternate between resources based on their motivation or the presence of higher-ranking individuals. The motivation to seek shade and water is also determined by the increase in the thermal load. In addition to individual motivation, another important factor influencing cow behavior is their gregarious nature. Cows are inherently social animals and tend to synchronize their activities, often performing the same behaviors at the same time [37]. However, this synchronization can be disrupted when resources are limited, leading individuals to reduce behavioral synchronization to avoid agonistic interactions. In our study, shade (10 m^2^ per cow) and water (one water trough for 22 cows) were not a limited resource. This suggests that the thermal environment emerged as a key factor modulating the influence of social hierarchy on resource use. For example, after prolonged periods without drinking or during high temperatures, cows show increased motivation to access water, leading to competition at the trough [38]. Although water was provided ad libitum in this study, unequal access can still occur if the trough is not appropriately designed or located, allowing social hierarchy to limit access for subordinate animals. While the effects of social hierarchy on food access are well documented and various mitigation strategies have been studied (e.g., barriers [39], space per animal [40]), little attention has been given to the shape, design, positioning, and location of water troughs [41,42]. Ensuring that these elements are carefully planned is crucial to prevent negative impacts on health, performance, and welfare, even in systems offering unrestricted water availability.

The highest frequency of grazing occurred during the initial observation hours, which fell within the comfort threshold for dairy cows. Grazing tends to be more intense in the early morning and late afternoon, coinciding with the cooler hours of day [43,44]. However, as environmental heat load increases, cows adjust their grazing behavior to reduce metabolic heat production, since more intense grazing can exacerbate thermal discomfort [20]. Along with grazing, cows also modified their rumination and idling behaviors in response to rising thermal stress. In our study, dominant cows more frequently idled and ruminated in the shade area, indicating a better use of this area to maintain thermal comfort and lose heat. Indeed, cows tend to prefer lying on cooler surfaces, such as shaded areas, likely benefiting from enhanced conductive heat transfer [45]. During periods of heat stress, cows can also spend more time standing, which increases the surface area exposed to airflow and facilitates heat dissipation through evaporative cooling, radiant heat loss, and convection [46,47]. Overall, our findings emphasize the importance of providing shade in pasture-based systems and suggest the need for adaptive strategies to optimize thermal comfort. Silvopastoral systems have the potential to create a comfortable environment for dairy cows, but effective environmental management is essential to maximize these benefits.

The reduction in visits to the water trough, alongside the increased use of shaded areas, can be partly explained by the location of the water trough, which remained in sunny areas. As the thermal challenge increased, cows adopted different behavioral strategies to maintain body homeostasis, with their movement and environmental exploration being influenced by both the thermal environment and social dynamics. When the thermal load exceeded the cows’ comfort thresholds, they tended to reduce their activity, preferring to stay in shaded areas [19]. This behavior likely serves as a strategy to minimize energy expenditure, redirecting metabolic resources toward heat dissipation through sensible and latent means. However, it is worth noting that locating the water trough within a paddock can facilitate access for lower-ranking cows (Coimbra et al. [16]). Social hierarchy exerts less negative influence when the water trough is located within a paddock, compared to outside [7]. The position of the water trough is important for ensuring water intake among animals, since subordinate cows can go up to 48 h without drinking if access is limited to once a day [47]. Nizzi et al. [38], monitoring individual drinking behavior of Holstein cows using electronic drinkers, also confirmed that social hierarchy can affect water access and trough visit duration, even when water availability is seemingly high. Burkhardt et al. [48], in a study with Brown Swiss cows, observed that lower-ranking cows often require multiple attempts to satisfy their water needs due to interruptions by high ranking animals. This observation resonates with our finding of higher water trough visit frequencies by lower-ranking cows, suggesting they might also be compensating for limited access, even if the large volume of our single trough may have mitigated direct competition. Beyond welfare concerns about restricted water access, this also compromises farm productivity, as herds with limited water access produce less milk [49]. Therefore, adequate structural planning of paddocks should integrate behavioral, social, and environmental aspects to promote equitable access to resources, with direct impacts on animal health, comfort, and performance.

While our study provides valuable insights into the effects of social hierarchy on the use of heat abatement resources by dairy cows, some limitations should be acknowledged. First, the experiment was conducted under specific climatic conditions, which may limit the generalizability of the findings to other geographic regions or housing systems. Second, our experiment was conducted under the standard conditions of a commercial farm, and it had to be integrated into the farm’s daily routine and utilize the existing infrastructure. Therefore, the number of animals in the lactating group, paddock dimensions, and water availability were not altered. Additionally, while behavioral budgets were measured, physiological indicators such body temperature, cortisol levels and nighttime behaviors were not assessed. Including these measures in future studies could provide a more comprehensive understanding of the broader implications for dairy cow performance and welfare. Future research should also investigate how shade availability interacts with multiple water troughs and assess the economic feasibility of such interventions for dairy farmers. Moreover, exploring the impact of social hierarchy on animal welfare indicators beyond thermoregulation, such as stress biomarkers and performance outcomes, would further support the development of precision climate control strategies. Finally, long-term studies conducted across diverse farm environments are necessary to better understand how social hierarchy influences the use of different heat abatement resources under varying conditions.

## 5. Conclusions

The frequency of visits to the water trough and the use of shaded areas were influenced by both social hierarchy and hours. Patterns of shade use varied across observation hours and social categories, with dominant cows more frequently engaging in lying idle and lying rumination. Sunny areas provide thermal challenges for dairy cows, resulting in prolonged exposure to discomfort. Our findings highlight the important role of silvopastoral systems in mitigating heat stress and providing comfortable areas (e.g., shaded areas) for dairy cows to engage in natural behaviors.

In the context of climate change, pasture-based dairy farms must ensure adequate availability of shade and easily accessible drinking water to minimize the effects of social competition, particularly under extreme heat conditions. The adoption of sustainable practices that promote thermal comfort can improve both animal welfare and productivity, while enhancing the resilience of production systems in the face of climate challenges. Silvopastoral systems, which integrate animal production with ecological restoration, offer the potential to reduce the impacts of extreme weather conditions at the farm level. Policymakers have a vital role in supporting initiatives that promote animal welfare in modern livestock production.

## Figures and Tables

**Table 1:** Average values (mean ± standard deviation) of age (years), weight (kg), and milk yield (L/day) by social category (dominant, intermediate and subordinate) in relation to the seasons (Autum, Winter, Spring and Summer).

**Autumn**					**Winter**			
**Social Category**	**Age**	**Weight**	**Milk Yield**		**Social Category**	**Age**	**Weight**	**Milk Yield**
Dominant (n = 5)	8.4 ± 4.2	460.8 ± 25.6	18.8 ± 2.9		Dominant (n = 6)	7.4 ± 2.8	441.8 ± 26.6	21.1 ± 2.3
Intermediate (n = 8)	5.3 ± 1.7	408.5 ± 51.1	15.9 ± 2.1		Intermediate (n = 9)	4.2 ± 1.9	380.1 ± 35.1	18.9 ± 2.6
Subordinate (n = 9)	4.1 ± 2.4	363.4 ± 29.3	15.5 ± 4.0		Subordinate (n = 7)	4.8 ± 2.8	364.2 ± 26.9	14.2 ± 2.1
**Spring**					**Summer**			
**Social Category**	**Age**	**Weight**	**Milk Yield**		**Social Category**	**Age**	**Weight**	**Milk Yield**
Dominant (n = 4)	6.7 ± 2.7	428.2 ± 22.7	22.1 ± 0.9		Dominant (n = 4)	6.5 ± 2.7	438.1 ± 19.5	15.4 ± 4.2
Intermediate (n = 9)	4.7 ± 1.4	388.8 ± 22.9	18.3 ± 3.5		Intermediate (n = 9)	5.3 ± 1.8	405.7 ± 29.2	14.8 ± 3.3
Subordinate (n = 9)	3.7 ± 1.4	364.1 ± 30.3	14.7 ± 3.2		Subordinate (n = 9)	3.6 ± 2.1	393.8 ± 38.4	15.3 ± 2.3

**Table 2:** Average values (mean ± standard deviation) of the variables air temperature (AT), relative humidity (RH), wind speed (WS), soil surface temperature (SST), black globe temperature (BGT), black globe-humidity index (BGHI), mean radiant temperature (MRT), and radiant heat load (RHL) in the different areas (shaded and sunny) of the silvopastoral system throughout the hours of the day.

Hours	AT (°C)				RH (%)				WS (m/s)				SST (°C)		
	Shaded	Sunny	*p*-Value		Shaded	Sunny	*p*-Value		Shaded	Sunny	*p*-Value		Shaded	Sunny	*p*-Value
08:00–08:55	15.9 ± 3.5	19.6 ± 4.2	0.01		88.6 ± 10.1	80.9 ± 12.9	0.20		0.50 ± 0.4	0.63 ± 0.5	0.23		16.6 ± 3.1	18.6 ± 4.3	0.20
09:00–09:55	18.8 ± 3.2	23.4 ± 3.4	<0.001		78.6 ± 13.3	66.8 ± 11.4	0.02		0.75 ± 0.4	1.00 ± 0.4	0.16		17.7 ± 3.1	23.6 ± 5.1	<0.001
10:00–10:55	21.4 ± 3.1	25.6 ± 2.7	<0.001		66.4 ± 11.8	57.4 ± 7.4	0.03		1.20 ± 0.6	1.62 ± 0.8	0.13		19.3 ± 4.1	29.3 ± 6.4	<0.001
11:00–11:55	23.1 ± 2.9	26.6 ± 2.6	<0.001		57.4 ± 10.4	51.6 ± 8.1	0.08		1.45 ± 0.6	1.95 ± 0.8	0.07		20.2 ± 3.6	33.1 ± 6.9	<0.001
12:00–12:55	24.1 ± 2.9	27.4 ± 2.5	<0.001		52.4 ± 9.5	49.0 ± 8.3	0.29		1.58 ± 0.9	2.00 ± 0.9	0.20		21.6 ± 4.2	35.1 ± 7.1	<0.001
13:00–13:55	24.9 ± 2.9	27.7 ± 2.6	0.01		49.4 ± 8.8	47.7 ± 7.9	0.59		1.53 ± 0.7	1.97 ± 0.7	0.08		22.6 ± 4.1	35.6 ± 7.2	<0.001
14:00–14:55	25.2 ± 2.9	28.0 ± 2.6	0.01		47.4 ± 8.3	46.5 ± 7.7	0.74		1.73 ± 0.6	2.08 ± 0.8	0.20		22.8 ± 3.9	33.3 ± 6.5	<0.001
**Hours**	**BGT (°C)**				**BGHI**				**MRT (W/m^2^)**				**RHL (W/m^2^)**		
	**Shaded**	**Sunny**	***p*-Value**		**Shaded**	**Sunny**	***p*-Value**		**Shaded**	**Sunny**	***p*-Value**		**Shaded**	**Sunny**	***p*-Value**
08:00–08:55	16.2 ± 3.8	23.9 ± 5.9	<0.001		62.3 ± 4.5	70.8 ± 6.8	<0.001		289.9 ± 4.5	304.3 ± 10.3	<0.001		401.7 ± 25.3	490.3 ± 65.8	<0.001
09:00–09:55	19.0 ± 3.5	29.4 ± 4.1	<0.001		65.4 ± 3.9	75.5 ± 4.7	<0.001		292.3 ± 4.9	314.4 ± 6.9	<0.001		417.6 ± 22.2	556.2 ± 48.8	<0.001
10:00–10:55	21.8 ± 3.6	31.7 ± 2.9	<0.001		68.3 ± 4.2	79.1 ± 3.4	<0.001		295.9 ± 4.4	319.6 ± 4.9	<0.001		435.9 ± 25.9	593.6 ± 35.9	<0.001
11:00–11:55	23.3 ± 3.2	32.4 ± 2.5	<0.001		69.7 ± 3.9	79.7 ± 3.1	<0.001		297.1 ± 3.8	322.4 ± 4.0	<0.001		442.9 ± 23.3	602.7 ± 29.8	<0.001
12:00–12:55	24.5 ± 3.2	33.1 ± 2.4	<0.001		70.9 ± 3.8	80.5 ± 2.9	<0.001		298.6 ± 3.9	327.1 ± 2.9	<0.001		451.9 ± 24.4	606.8 ± 21.9	<0.001
13:00–13:55	25.5 ± 3.3	33.4 ± 2.5	<0.001		72.1 ± 4,1	80.8 ± 3.1	<0.001		300.3 ± 5.1	324.2 ± 3.2	<0.001		462.3 ± 31.6	608.2 ± 24.3	<0.001
14:00–14:55	25.6 ± 3.2	33.5 ± 3.8	<0.001		72.2 ± 3.9	80.9 ± 3.1	<0.001		299.7 ± 3.9	322.0 ± 2.9	<0.001		458.4 ± 23.9	607.7 ± 22.5	<0.001

## Data Availability

The original contributions presented in this study are included in the article. Further inquiries can be directed to the corresponding author.
